# A Novel Topology of a 3 × 3 Series Phased Array Antenna with Aperture-Coupled Feeding

**DOI:** 10.3390/s24186128

**Published:** 2024-09-23

**Authors:** Guang Yang, Choon Sae Lee, Linsheng Zhang

**Affiliations:** Department of Electrical and Computer Engineering, Southern Methodist University, Dallas, TX 75205, USA; csl@lyle.smu.edu (C.S.L.); linshengz@mail.smu.edu (L.Z.)

**Keywords:** phased array antenna, series aperture coupled, microstrip antenna, power splitter, quadrature hybrid

## Abstract

This paper presents a novel 3 × 3 phased array antenna optimized for 4 GHz operation, achieving a realized gain of 13.2 dBi and enabling 30-degree beam steering with a minimal capacitance variation of 1.5 pF. The design features a series aperture-coupled feeding mechanism that not only reduces the antenna’s size but also simplifies the fabrication process, making the device both cost-effective and compact. Integrating cost-efficient quadrature-hybrid phase shifters and novel power splitters with cascaded quadrature hybrids ensures uniform power distribution and precise beam steering. The innovative use of these components addresses common challenges in phased array systems, such as space constraints, high costs, and complex power distribution.

## 1. Introduction

Phased array antennas are integral to a diverse array of applications spanning both civil and military sectors. These antennas leverage electronically steerable beams to enhance communication, radar, and electronic warfare capabilities. However, the state-of-the-art designs of contemporary phased array antennas are often encumbered by intricate architectures and substantial physical bulk [1,2,3]. This complexity is exacerbated by prohibitive costs, which impinge upon their widespread adoption and deployment across various scenarios. A critical facet of phased array design is the precise allocation of power and phase to each individual radiating element. Traditional power splitting methods, such as quarter-wavelength transformers [4,5] and Wilkinson power splitters [6], face increasing challenges due to their inherent limitations in large array configurations. These limitations include difficulties in managing complex network topologies and significant power dissipation, which can undermine the system’s overall efficiency. The complexity of power distribution grows exponentially with the number of array elements. Specifically, the requirement for thin transmission lines to achieve uniform field distribution further exacerbates the challenges. Thinner transmission lines introduce several issues, including elevated radiation losses, diminished power handling capacities, increased mechanical fragility, and heightened susceptibility to environmental damage. Additionally, the manufacturing process for these components becomes significantly more challenging, complicating the fabrication of such advanced phased array systems.

In light of these challenges, this paper presents an innovative approach to phased array antenna design aimed at simplifying fabrication and reducing costs. The proposed solution involves a series aperture-coupled feeding mechanism, which not only minimizes the overall antenna size but also streamlines the design process. Furthermore, we introduce novel power splitters incorporating cascaded quadrature hybrids, which contribute to a compact and efficient antenna architecture. By adjusting the stub length in the power divider, this method facilitates the precise achievement of desired power levels at the output ports, circumventing the need for complex design and fabrication processes that are particularly challenging when high power division ratios are required. Additionally, the phase shifters employed in the proposed phased array utilize quadrature hybrids to facilitate a broad range of beam steering capabilities [7,8]. This design choice ensures that the resultant antenna structure remains cost-effective and exhibits minimal insertion loss. The integration of these advancements aims to overcome the traditional limitations of phased array systems, making them more accessible and practical for a broader range of applications. Through these innovations, we anticipate significant improvements in both the performance and affordability of phased array antennas, addressing critical needs in contemporary and future technological landscapes.

## 2. Antenna Configuration and Design

### 2.1. Antenna Configuration

**Figure 1:**
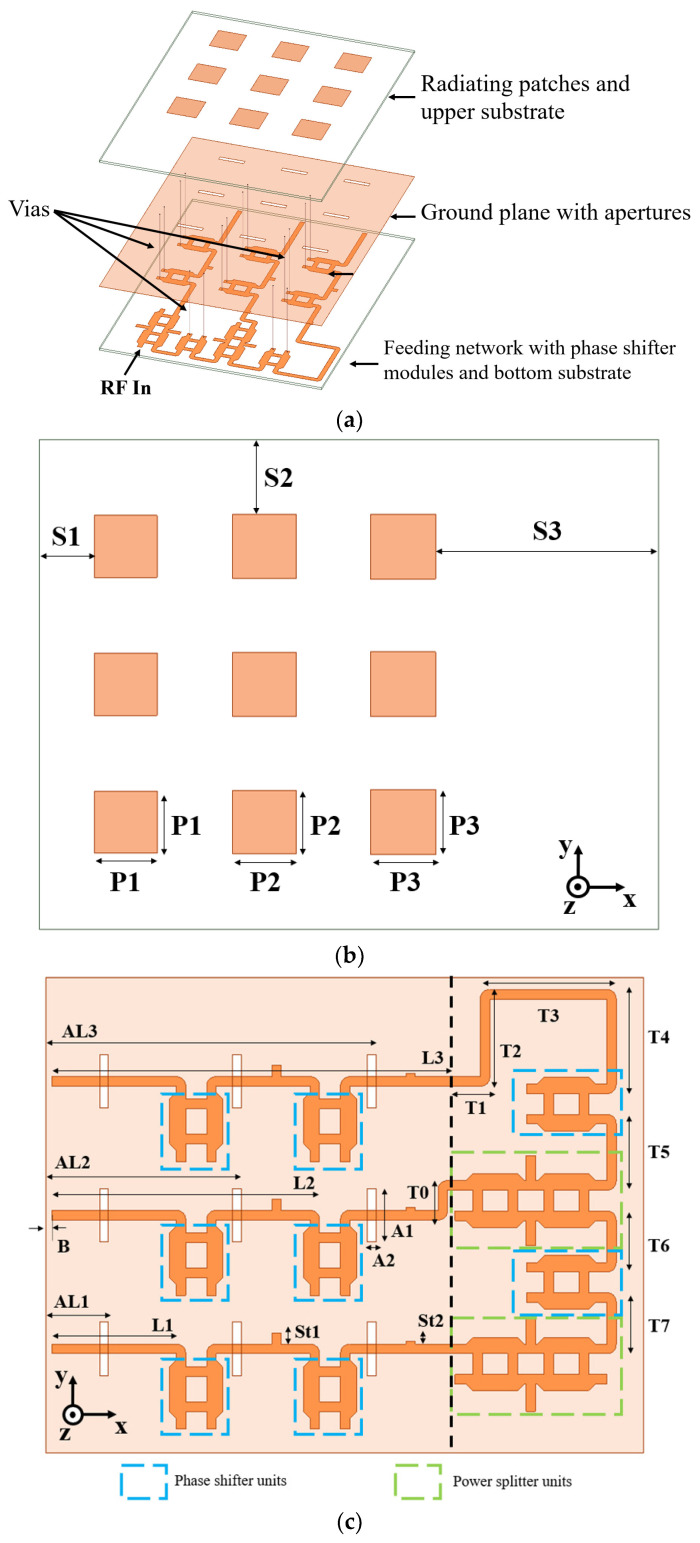
Proposed 3 × 3 phased array antenna: (**a**) exploded view, (**b**) top view of the radiating patches and upper substrate, and (**c**) top view of the lower substrate with a feeding network.

Figure 1 illustrates the configuration of the proposed phased array antenna, comprising three layers. The antenna employs Rogers RO4003C substrates with a dielectric constant of 3.55 and a loss tangent of 0.0027 for the top and bottom layers, each with a thickness of 62 mils. The radiating elements are placed on the upper surface of the top substrate, while the ground plane with coupling apertures is on the top side of the lower substrate. The feeding network, along with the phase shifter units, is situated on the bottom side of the lower substrate. All layers are securely fastened using nylon screws. The dimensions of the upper and lower substrates are 201 mm × 160 mm, and the inter-element spacing is 45 mm. Other parameters are listed in Table 1.

The design process is optimized by breaking down the entire structure into three 1 × 3 subarrays. Each subarray undergoes three distinct design phases: configuring the radiating elements, developing the feed network, and integrating the phase shifters. After completing the design of each subarray, the three subarrays are assembled to construct the full planar phased array.

### 2.2. Radiating Elements

**Figure 2:**
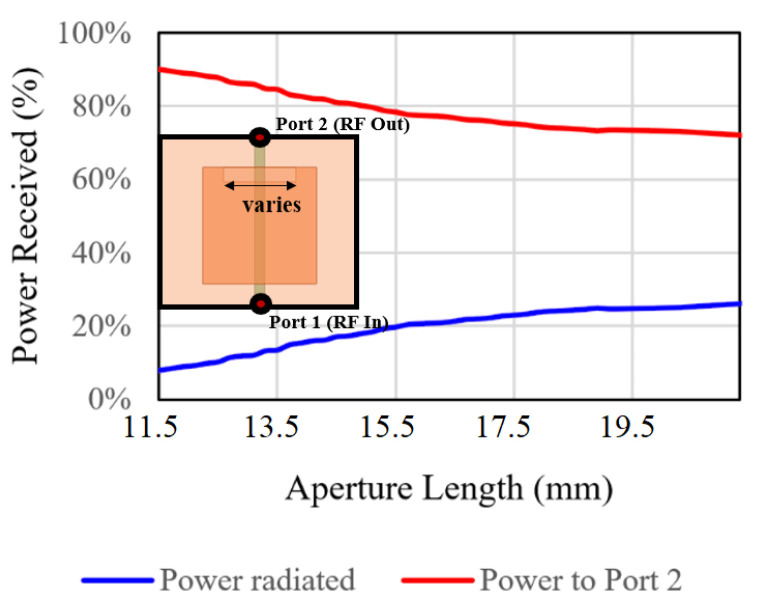
Radiated power is saturated to a certain value as the aperture length increases.

The position of the aperture in an aperture-coupled patch antenna determines the input impedance, while its length dictates how much power radiates [9]. The longer the aperture is, the more the antenna radiates. However, the radiated power levels off as the aperture length increases, as shown in Figure 2, which illustrates the power distribution for different aperture lengths in a 22 mm × 22 mm radiating element of an antenna array with a 3 mm aperture width. The substrates are Rogers 4003C with a thickness of 60 mils.

**Figure 3:**
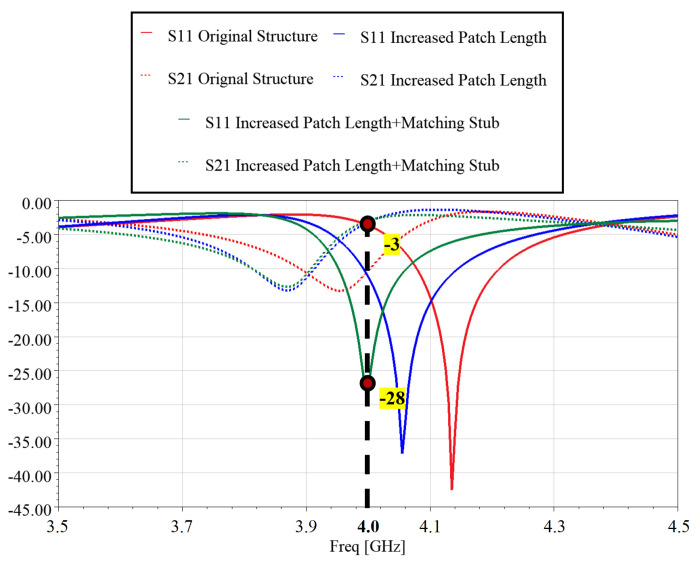
Different techniques used to design a single aperture coupled element integrated with a 1:1 power splitter.

To overcome such a shortcoming, a stub at the feed transmission line is used to achieve a specified radiated power for each patch element beyond the otherwise limited level while minimizing signal reflection. While designing each element, the patch size [10] has to be adjusted to compensate for any frequency shift, as shown in Figure 3. The figure shows transmission performances for different techniques used to design a single aperture-coupled element with a 1:1 power divider at 4.0 GHz.

**Figure 4:**
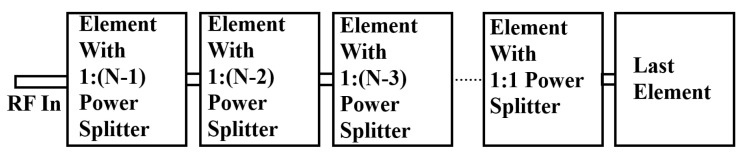
1 × N subarray structure with the proposed series aperture-coupled feeding network.

The proposed design can be used to construct a series feeding network for a linear array. This design approach offers a straightforward solution for creating an array structure that is easily scalable up to a 1 × N array, as depicted in Figure 4. In order to assure the same radiated power for all elements, each element has its own unique ratio of the radiated power over the transmitted power [10].

**Figure 5:**
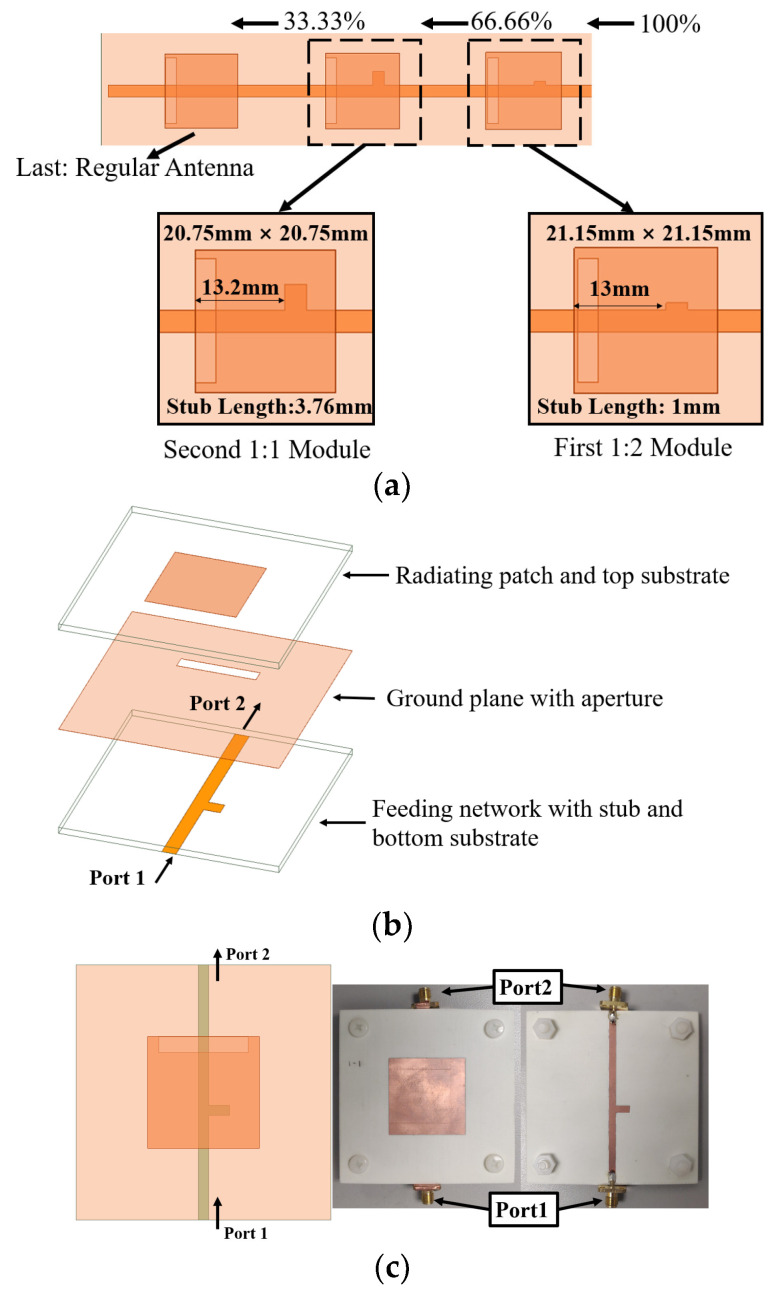
The module’s breakdown and analysis: (**a**) subarray analysis, (**b**) experimental setup of proposed aperture coupled structure in exploded view, and (**c**) top view of one of the modules.

To simplify the analysis, the subarray of 1 × 3 is divided into three modules, as shown in Figure 5a. The first and second modules both consist of a radiating element along with a transmission line that includes a matching stub. The third module adheres to the conventional design of an aperture-coupled antenna. Table 2 offers a comprehensive summary of the transmission performances across all modules.

### 2.3. Phase Shifter

**Figure 6:**
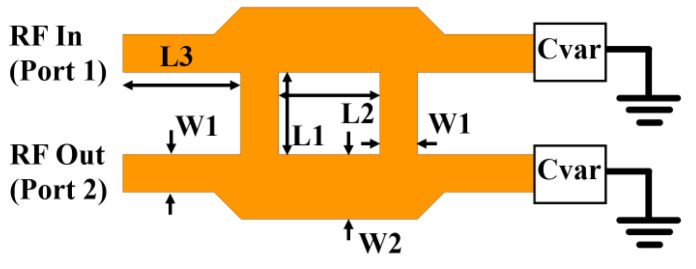
The structures of the proposed phase shifter.

In the proposed system, each phase shifter employs a reflection-type quadrature hybrid coupler. Two capacitors (Knowles Voltronics JZ030 [11]) are arranged in series with the coupler to function at the operational frequency of 4 GHz, as depicted in Figure 6 (parameters: W1 = 3.2 mm, W2 = 5.5 mm, L1 = 7 mm, L2 = 8.6 mm, L3 = 10 mm). The variable capacitances allow for the fine-tuning of the phase shift by altering the reactance in the signal path [9,12]. Owing to the unique characteristics of the quadrature hybrid, this type of phase shifter offers precise phase shifting while maintaining constant amplitude, good isolation, and low insertion loss.

**Figure 7:**
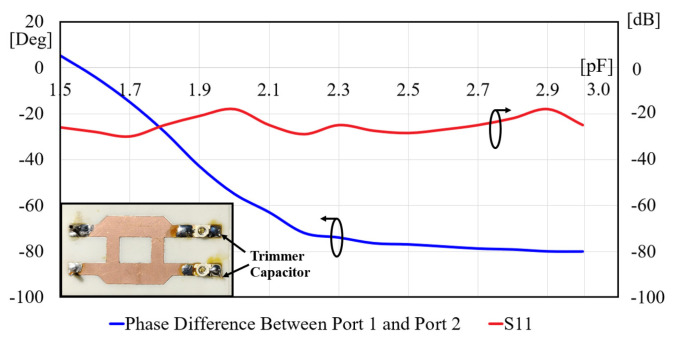
Measured phase shift and transmission of the quadrature-hybrid phase shifter as a function of capacitance at 4 GHz.

Figure 7 illustrates the measured phase shift as a function of capacitance. It is noteworthy that even a slight capacitance variation of 1.5 pF induces a substantial phase shift of 91 degrees.

### 2.4. Integration

**Figure 8:**
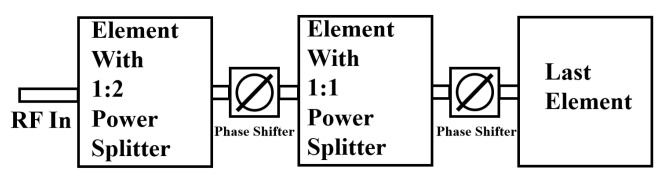
1 × 3 phased array with proposed series aperture-coupled feeding network.

An efficient beamforming subarray is achieved by integrating quadrature-hybrid phase shifters into the radiating array structure, as shown in Figure 8. Each phase shifter between different modules provides a phase shift from 9.2 degrees to −79.1 degrees with a variation of capacitance from 1.5 pF to 3 pF for beam steering from −15 to +15 degrees. All phase shifters are uniform, featuring only one input signal for each beam direction.

### 2.5. Power Splitter

**Figure 9:**
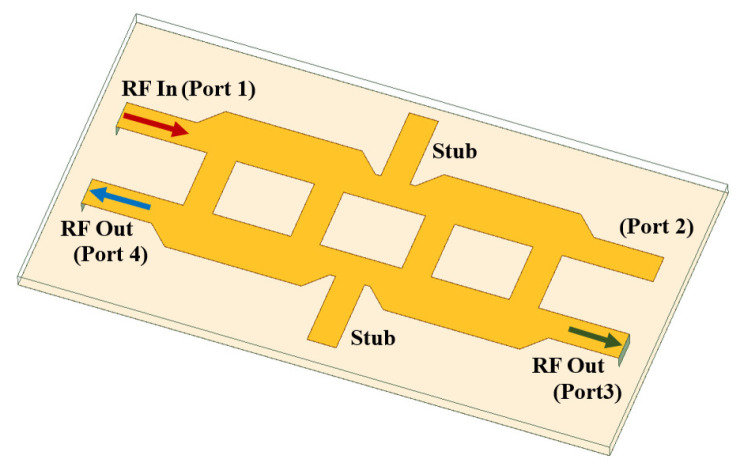
Proposed power splitter structure.

A novel power splitter with quadrature hybrids is placed between neighboring subarrays. The proposed power splitter, as shown in Figure 9, is a sequential connection of quadrature hybrids complemented by additional stubs. Such an arrangement enables accurate control over power division. Port 1 functions as the input, Port 2 is isolated, while Port 3 and Port 4 are outputs after the required power splitting.

It is well known that the four ports of a single coupler have the following amplitudes [7]:

(1) $$B_{1}=0\;B_{2}=-\frac{j}{\sqrt{2}}\;B_{3}=-\frac{1}{\sqrt{2}}\;B_{4}=0$$

**Figure 10:**
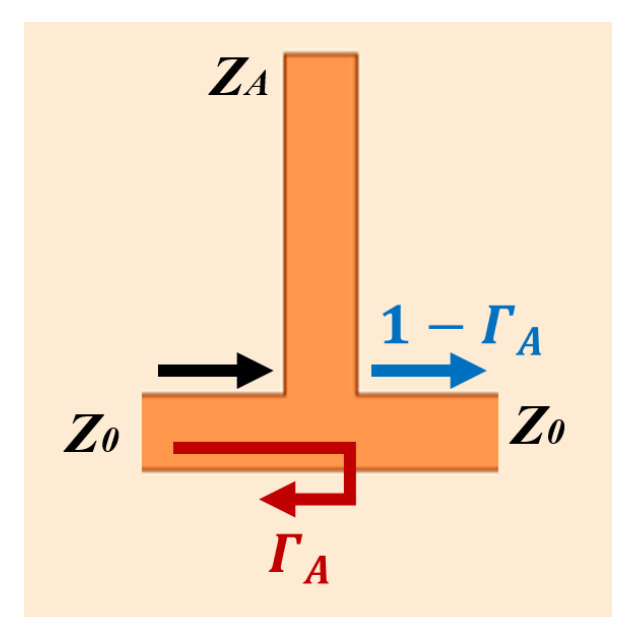
A stub introduces reflection.

The impedance *Z_A_* of the stubs between two couplers (as shown in Figure 10) is

(2) $$Z_{A}={-Z}_{0}jcot\left(\beta l\right)$$

where *Z*_0_, *β*, and *l* are the characteristic impedance, the phase constant, and the physical length of the transmission line, respectively.

It is shown that the amplitudes at the four ports of the proposed power splitter are

$$D_{1}\left(Port1\right)=\left(-\frac{j}{\sqrt{2}}\Gamma_{A}\right)\left(-\frac{j}{\sqrt{2}}\right)+\left(-\frac{1}{\sqrt{2}}\Gamma_{A}\right)\left(-\frac{1}{\sqrt{2}}\right)=0$$

$$D_{2}\left(Port2\right)=\left(-\frac{j}{\sqrt{2}}(1-\Gamma_{A})\right)\left(-\frac{j}{\sqrt{2}}\right)+\left(-\frac{1}{\sqrt{2}}(1-\Gamma_{A})\right)\left(-\frac{1}{\sqrt{2}}\right)=0$$

$$D_{3}\left(Port3\right)=\left(-\frac{j}{\sqrt{2}}(1-\Gamma_{A})\right)\left(-\frac{1}{\sqrt{2}}\right)+\left(-\frac{1}{\sqrt{2}}(1-\Gamma_{A})\right)\left(-\frac{j}{\sqrt{2}}\right)=j(1-\Gamma_{A})$$

$$D_{4}\left(Port4\right)=\left(-\frac{1}{\sqrt{2}}\Gamma_{A}\right)\left(-\frac{j}{\sqrt{2}}\right)+\left(-\frac{j}{\sqrt{2}}\Gamma_{A}\right)\left(-\frac{1}{\sqrt{2}}\right)=j\Gamma_{A}$$

where

(3) $$\Gamma_{A}=\frac{Z_{0}-Z_{0}\|Z_{A}}{Z_{0}+Z_{0}\|Z_{A}}$$

**Figure 11:**
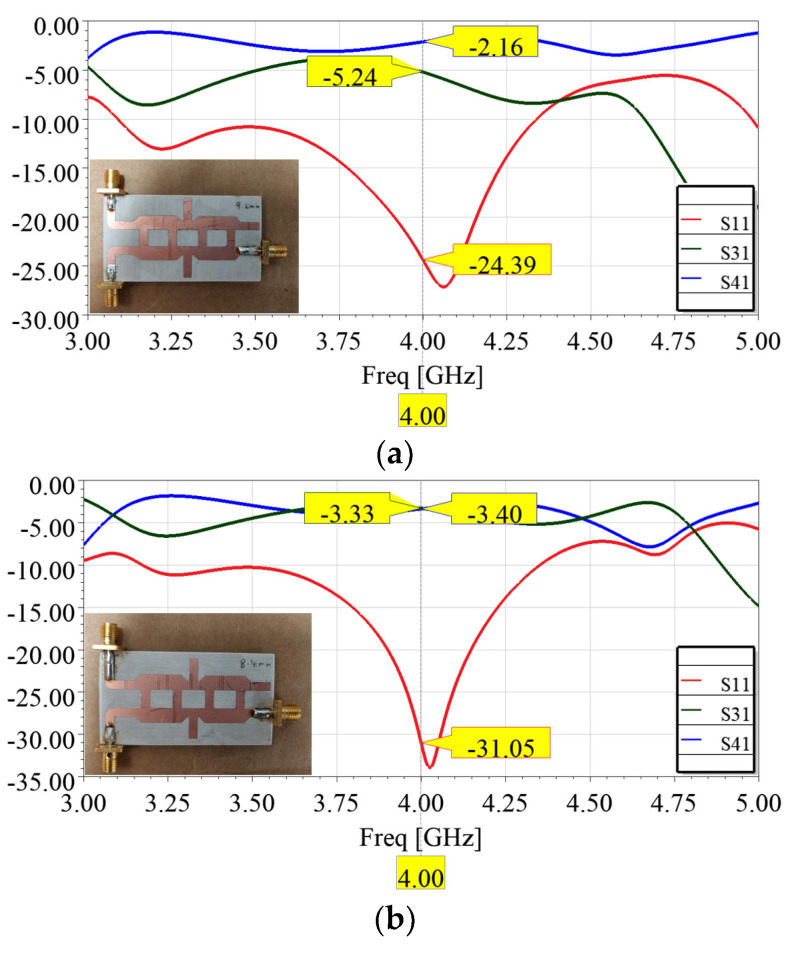
The measured scattering parameter of (**a**) 1:2 power splitter with a stub length of 9.2 mm, and (**b**) 1:1 power splitter with a stub length of 8.4 mm.

The isolation of Ports 1 and 2 is apparent, allowing for unimpeded signal transmission. Meanwhile, Ports 3 and 4 offer the flexibility to split the power from Port 1 to any desired level by varying the stub length. Figure 11 shows the prototypes and their performances with the proposed power splitters.

### 2.6. Mechanism of Phased Array

**Figure 12:**
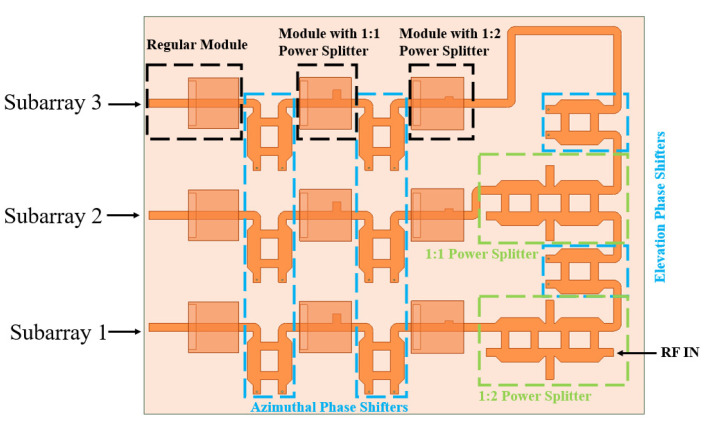
The operational procedure of the phased array system.

In the operational process depicted in Figure 12, the input port feeds the 1 × 3 subarrays with equal power allocation. The first subarray receives one-third of the total input power, while the remaining two-thirds transmits to the next stage via a 1:2 power splitter. Subsequently, after passing through the first phase shifter for elevation beam steering, the remaining two-thirds of the power is divided again into two equal portions by a 1:1 power splitter. Half of this divided power is allocated to the second subarray while the other half is forwarded to the final subarray, thus ensuring all three subarrays receive the same amount of power.

Each subarray comprises three modules. Integrating power splitters within these modules ensures even power distribution among the individual elements. Ultimately, this design scheme guarantees uniform power distribution across all nine elements.

## 3. Numerical and Experimental Results

**Figure 13:**
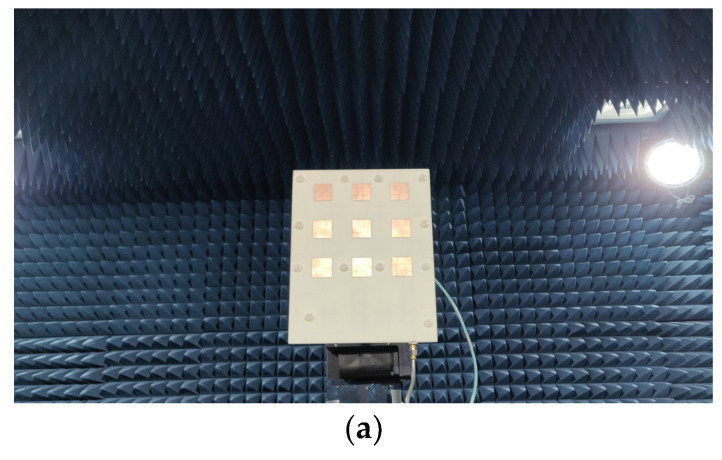

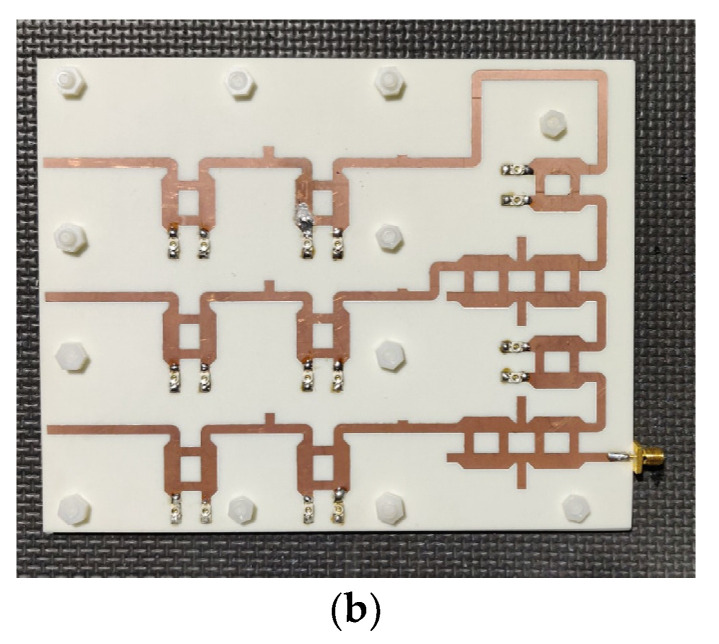
The proposed antenna under test: (**a**) the front side with radiating patches and (**b**) the back side with the feeding network.

**Figure 14:**
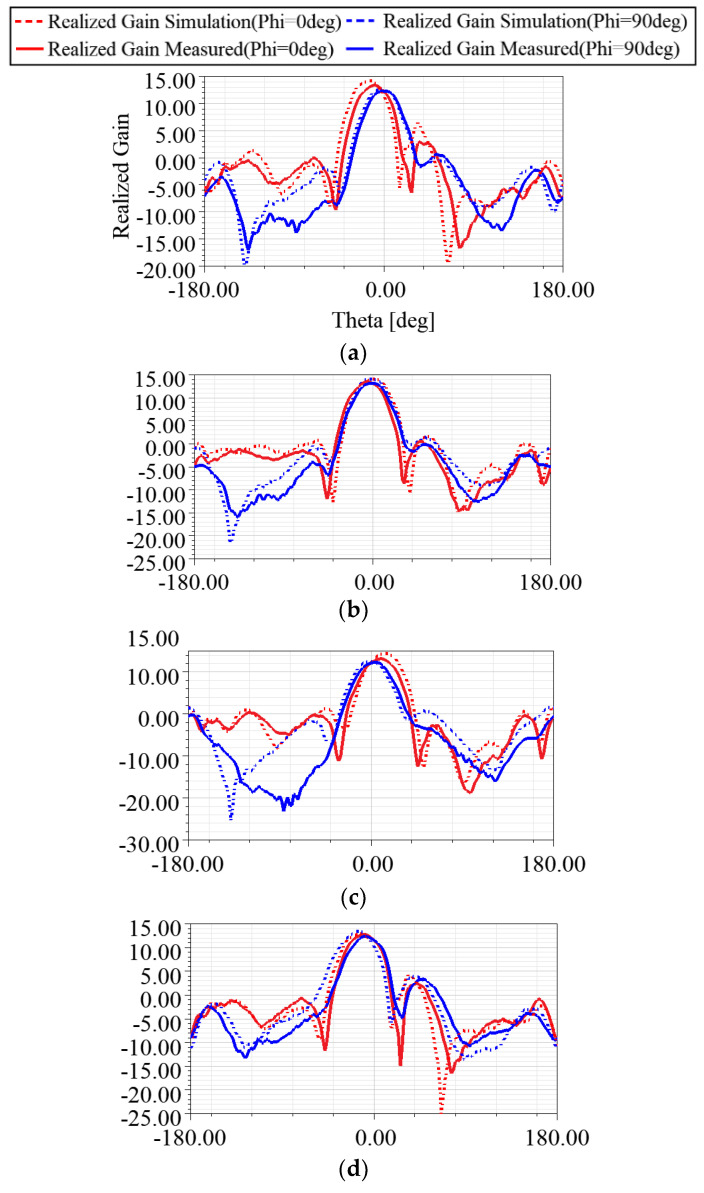

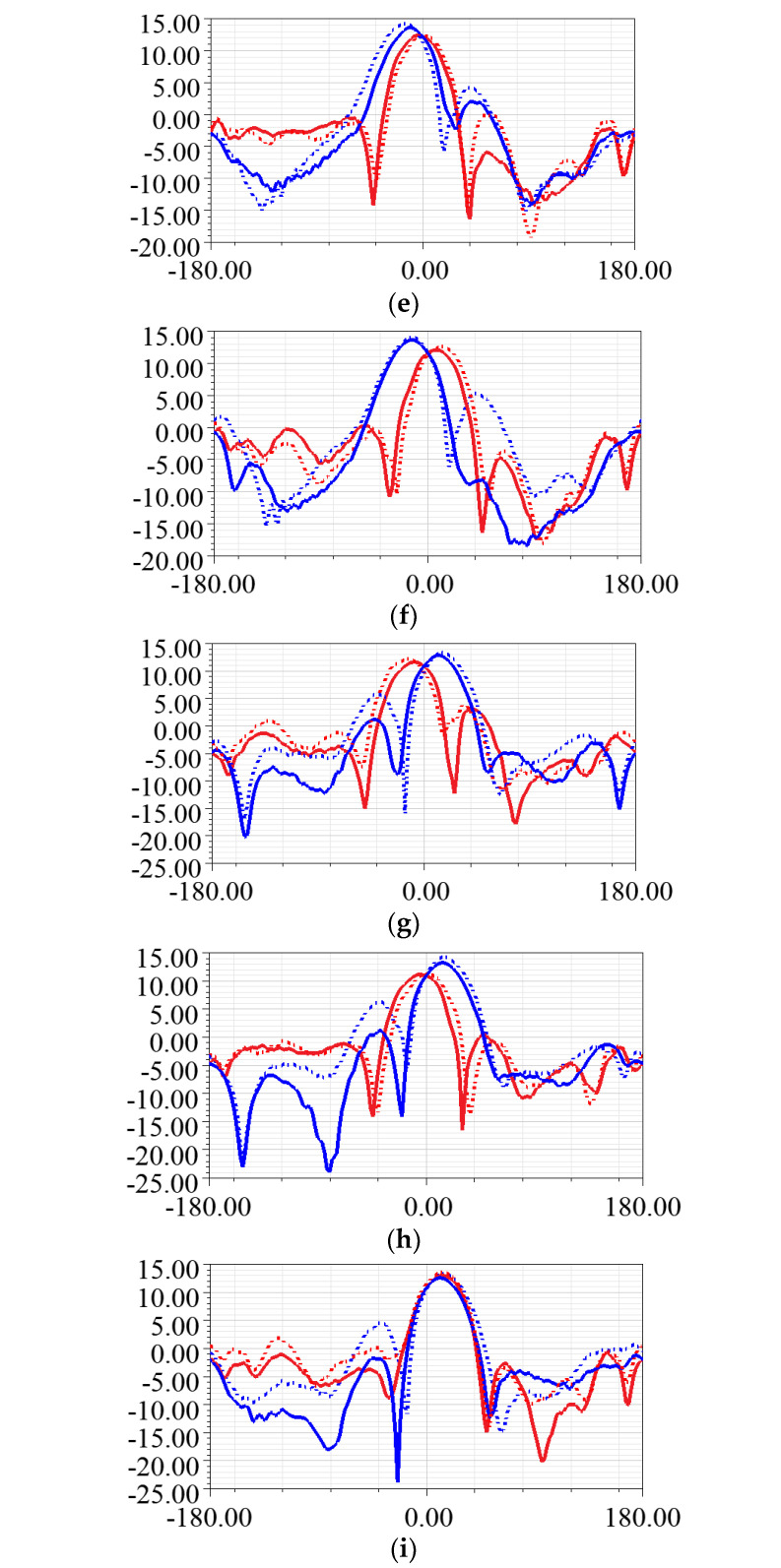
Comparing the radiation patterns between simulation and measured data under various conditions. (**a**) Azimuthal phase shifters with a capacitance value of 1.5 pF and elevation phase shifters with a capacitance value of 2.0 pF. (**b**) Azimuthal phase shifters with a capacitance value of 2.0 pF and elevation phase shifters with a capacitance value of 2.0 pF. (**c**) Azimuthal phase shifters with a capacitance value of 3.0 pF and elevation phase shifters with a capacitance value of 2.0 pF. (**d**) Azimuthal phase shifters with a capacitance value of 1.5 pF and elevation phase shifters with a capacitance value of 3.0 pF. (**e**) Azimuthal phase shifters with a capacitance value of 2.0 pF and elevation phase shifters with a capacitance value of 3.0 pF. (**f**) Azimuthal phase shifters with a capacitance value of 3.0 pF and elevation phase shifters with a capacitance value of 3.0 pF. (**g**) Azimuthal phase shifters with a capacitance value of 1.5 pF and elevation phase shifters with a capacitance value of 1.5 pF. (**h**) Azimuthal phase shifters with a capacitance value of 2.0 pF and elevation phase shifters with a capacitance value of 1.5 pF. (**i**) Azimuthal phase shifters with a capacitance value of 3.0 pF and elevation phase shifters with a capacitance value of 1.5 pF.

The measurements were performed in the SMU antenna anechoic chamber using the option of far-field scanning the Allwave antenna measurement system, as shown in Figure 13. Three cases were examined to assess the impact of varying capacitance values on the phase shift of each phase shifter. The measured phase shifts were found to be 9.2 degrees, −47.8 degrees, and −79.1 degrees for the capacitances of 1.5, 2.0, and 3.0 pF, respectively, as shown in Figure 7. Figure 14 illustrates the measured radiation patterns of the beam directions in comparison with the simulation. The antenna maintained a return loss of approximately −18 dB across varying capacitance values. The excellent agreement between the theory and the experiment validates the proposed concept of electronic beam steering.

The simulation indicated a beam tuning range of ±15 degrees, while the measurements showed a slightly narrower range of ±14.5 degrees for both E-plane and H-plane. Table 3 presents a comparative analysis between the proposed structure and other documented 3 × 3 designs in the existing literature. References [13,14] introduce a conventional feeding network, resulting in a complex system that requires assigning individual RF inputs to each radiating patch for beam steering, while in [15,16], the number of RF inputs is reduced but multiple input feeds are still necessary. Moreover, as for the efficiency, the proposed structure capitalizes on the advantages of the quadrature hybrid structure, leveraging its excellent isolation properties to minimize signal leakage and ensure signal transmission with minimum loss. By fully utilizing the available space, all components are efficiently integrated to provide substantial antenna gain, thereby establishing an effective feeding topology.

## 4. Conclusions

This paper presents a comprehensive and innovative approach to phased array antenna design, one specifically tailored for 4 GHz operation with advanced beam steering capabilities. The proposed antenna structure successfully addresses several key challenges associated with traditional phased array systems, including complex architectures, substantial physical bulk, and high costs. By integrating a series aperture-coupled feeding mechanism and cascaded quadrature hybrid power splitters, the design not only minimizes the overall antenna size but also simplifies the fabrication process, making the antenna more accessible and cost-effective. The inclusion of quadrature hybrid coupler phase shifters further enhances the antenna’s functionality, enabling precise and uniform beam steering within a compact form factor. The experimental results validate the effectiveness of the proposed design, demonstrating excellent agreement with theoretical predictions and showcasing the antenna’s ability to achieve beam steering over a range of ±14.5 degrees. The innovative power distribution and phase shifting techniques employed in this design ensure efficient power management and minimal insertion loss, which are critical for the performance of phased array systems. Compared to existing designs, the proposed structure offers significant improvements in terms of simplicity, efficiency, and integration, making it a highly viable solution for wireless applications where space, environmental, and cost constraints are prevalent.

## Figures and Tables

**Table 1:** Dimensions of the proposed structures.

Parameter	Value (mm)	Parameter	Value (mm)
P1	20.30	T7	14.00
P2	20.75	A1	18.00
P3	21.15	A2	3.00
S1	17.85	L1	45.00
S2	24.85	L2	90.00
S3	72.425	L3	135.00
T0	13.00	AL1	21.00
T1	13.00	AL2	65.70
T2	32.50	AL3	111.00
T3	45.00	St1	3.76
T4	32.00	St2	1.00
T5	18.00	B	2.00
T6	14.00		

**Table 2:** Module transmission performance.

Module# (From the Input)	Return Loss (Sim./Meas.,dB)	Power Transmitted (Sim./Meas.,dB)	Power Radiated (Sim./Meas.,dB)
**First**	−29.95/−26.70	−1.76/−1.78	−4.77/−4.73
**Second**	−29.25/−27.22	−3.00/−2.98	−3.01/−3.04
**Third (Last)**	−30.48/−28.35	NA	−0.01/−0.03

**Table 3:** Comparison of proposed and reported designs.

Feature	[13]	[14]	[15]	[16]	This Work
**Operating Frequency (GHz)**	12	5.2	28	9.65	4
**No. of Input Port**	9	36	4–6	6	1
**No. of Element**	3 × 3	6 × 6	3 × 3	3 × 3	3 × 3
**Boresight Gain (dBi)**	10.45	18.1	14.9	12.5	13.2
** $\mathrm{Array}\;\mathrm{Size}\;(\lambda_{0}^{2})$ ** *	2 × 2 (phased array system not included)	3 × 3 (phased array system not included)	2 × 2 (phased array system not included)	3.26 × 3.26 (phased array system not included)	2.68 × 2.13 (phased array system included)

* *λ*_0_ represents the free-space wavelength at operating frequency.

## Data Availability

Data are contained within this article.
